# Impact of Anionic Substitution in Yb_14_MgSb_11–*x*_As_*x*_ Compounds on the Electronic and Thermoelectric Properties

**DOI:** 10.1021/acs.jpcc.2c05597

**Published:** 2022-10-20

**Authors:** Trinh Vo, Paul von Allmen, Dean Cheikh, Sabah Bux, Jean-Pierre Fleurial

**Affiliations:** Jet Propulsion Laboratory, California Institute of Technology, Pasadena, California91109, United States

## Abstract

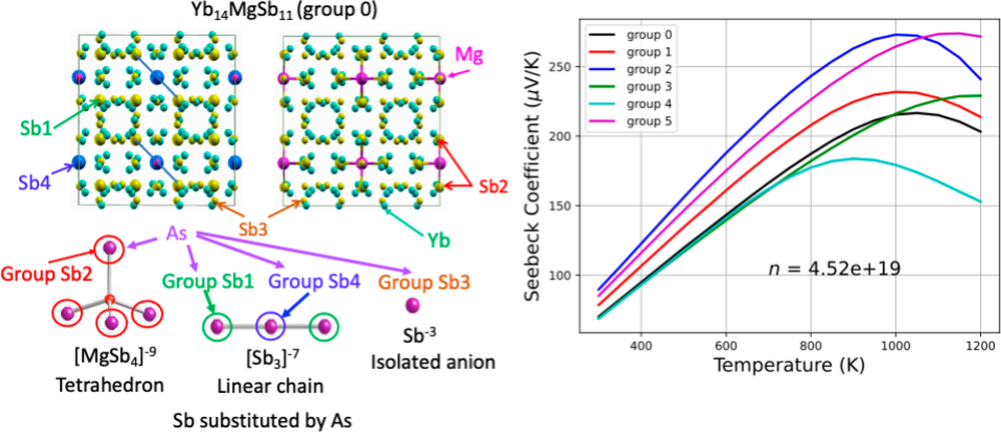

The effects of anionic site substitution on the electronic transport properties of Yb_14_MgSb_11–*x*_As_*x*_ compounds were investigated using density functional theory (DFT) with on-site Coulomb interaction correction (PBE+U). By replacing the Sb atoms at the four symmetry sites in Yb_14_MgSb_11_ with As, we found that the electronic and thermoelectric properties of the compound can be altered substantially. For most of the cases, the thermoelectric properties improve compared to the base compound Yb_14_MgSb_11_. Substitution at the tetrahedral site (Sb2) in particular yields the highest improvement in the thermoelectric properties. Detailed insight into the electronic and structural changes caused by the selective site substitutions is also discussed.

## Introduction

Thermoelectric generators (TGs) are devices that are routinely used to convert waste heat into electric power. Their application has recently become more relevant and widespread as the desire to reduce the emission from greenhouse gases has increased. In particular, in deep space missions, where radioisotope materials are used as the main heat source, radioisotope thermoelectric generators (RTGs) have proven to be efficient converters of heat into electric power when solar energy would be ineffective or insufficient. Examples of such applications include the successful use of RTGs in NASA missions to Mars and Jupiter. The efficiency of a TG or RTG is determined partially by the figure of merit *ZT* of the materials in the device
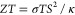
1where *S* is the Seebeck coefficient, σ is the electrical conductivity, and κ is the thermal conductivity, which includes electronic *κ*_e_ and lattice *κ*_l_ contributions with κ = *κ*_l_ + *κ*_e_. While *κ*_e_ mainly depends on the electronic band structure (through a dependence on the mobility, which is inversely proportional to the mass of the charge carriers), *κ*_l_ is determined by the phonon dispersion and phonon scattering. Optimization of the figure of merit for a homogeneous material has shown to be difficult. Large *ZT* values require a large *S*, high σ, and low κ, but an increase in σ is accompanied by an increase in *κ*_e_, following the Wiedemann–Franz law.

For several years, considerable effort has been devoted to the search for compounds with higher *ZT*. However, obtaining values of *ZT* higher than 1 is still challenging. Theoretical and experimental studies in low-dimensional thermal electric materials such as nanowires, quantum wells, and quantum dots can improve *ZT* signinficantly.^1−5^ However, in practice, the use of these nanomaterials is still limited due to difficulties in fabrication, processing, and obtaining the desired mechanical strength, leading to low overall device efficiencies. On the other hand, bulk materials remain more advantageous for practical device applications and have recently become the focus of many research activities, as the value of ZT can be improved considerably in materials with complex structures. Examples of such materials include clathrates, zintls, chalcogenides, and skutterudites.^6−11^ One of the advantages of structural complexity is that doping with defects not only reduces the thermal conductivity through scattering but also yields favorable electronic properties. Furthermore, the carrier concentration can be varied through doping, alloying, or controlling the concentration of vacancies, making the materials either a semiconductor or a weak metal and thus facilitating optimization of *ZT*.

A large body of published experimental work is already available for those materials.^6−11^ In particular, the discovery of the zintl 14–1–11 compound Ca_14_AlSb_11_ has spurred considerable interest in studies of high-temperature thermoelectric materials. Having a complex structure, several derivatives of Ca_14_AlSb_11_ can be prepared by substitution on the cationic, anionic, or metal sites. Modification of the chemical composition of the zintl materials can change the structural, electronic, magnetic, and hence thermoelectric properties to a variable degree.^12−14^ Among these derivatives, Yb_14_MnSb_11_ has attracted considerable attention due to its high figure of merit (*ZT* ∼ 1.4) at high temperature.^15,16^ At the same time, further experimental studies on other derivatives of Yb_14_MnSb_11_ were conducted in the hope of improving *ZT*, but the improvement has been modest,^17−22^ especially at temperatures larger than 1200 K. However, the origin of the difficulties in further improving *ZT* in Yb_14_MnSb_11_ derivatives has not been studied thoroughly and systematically. In addition, due to the presence of Mn (an element that is well-known for its complex magnetic properties), the magnetic properties of Yb_14_MnSb_11_ can influence the thermoelectric properties in complicated ways, since Yb_14_MnSb_11_ is ferromagnetic up to 56 K.^23−27^ On the other hand, an alternative to Yb_14_MnSb_11_, Yb_14_MgSb_11_, was recently synthesized in the laboratory and was found to feature a *ZT* close to that of Yb_14_MnSb_11_. The use of Yb_14_MgSb_11_ helps to avoid the complications caused by the magnetic element Mn present in Yb_14_MnSb_11_. While extensive experimental effort was undertaken to explore the compositional parameter space of thermoelectric materials by site substitutions in Yb_14_MnSb_11_, only little experimental work is available on substitutions in Yb_14_MgSb_11_.^28−31^ Additionally, due to the complex nature of the chemistry in zintl materials, insight and systematic understanding of the role of selective substitution at each site in the zintl compounds on the electronic properties is still lacking. Theoretical investigation into these effects is necessary because it supports and accelerates the experimental search for new thermoelectric materials through band structure engineering. To our knowledge, the study of the substitution on the anionic sites in Yb_14_MgSb_11_ has not yet been reported. In this work, we systematically study the effects of substituting Sb with As atoms in Yb_14_MgSb_11_ on the electronic and thermoelectric properties of Yb_14_MgSb_11–*x*_As_*x*_ compounds, using first-principles computation. The study also sheds light on the thermoelectric properties of the derivatives of Yb_14_MnSb_11_.

The paper is organized as follows. In the Methods section, we describe the computational details of the systems Yb_14_MgSb_11–*x*_As_*x*_. In the Results and Discussion section, we discuss the results by dividing them into three parts: (i) electronic properties, (ii) transport properties, and (iii) heat of formation.

## Methods

The structural relaxation and electronic properties of Yb_14_MgSb_11–*x*_As_*x*_ compounds are computed with the open source DFT software package Quantum Espresso (QE).^32−34^ The PBE-type generalized gradient approximation (GGA)^34^ and PBE plus on-site Coulomb interaction (PBE+U)^35−38^ were used and compared. Vanderbilt ultrasoft pseudopotentials^39^ are used for Yb, Mg, Sb, and As. For Yb, the pseudopotential was built with the 4f electrons included in the valence states (6s^2^ 5d^0^ 4f^14^). An energy cutoff of 50 Ry for the wave functions and a charge density cutoff of 400 Ry are used. The Mazari–Vanderbilt smearing scheme^40^ is used to speed up the convergence toward self-consistency. A smearing value of ∼0.136 eV and a Brillouin zone *k*-point sampling of 6 × 6 × 6 and 15 × 15 × 15 are used for the relaxation and transport property calculations, respectively. The choice of these parameters results from detailed convergence tests on the density of states (DOS) and Seebeck coefficient calculations.

**Figure 1:**
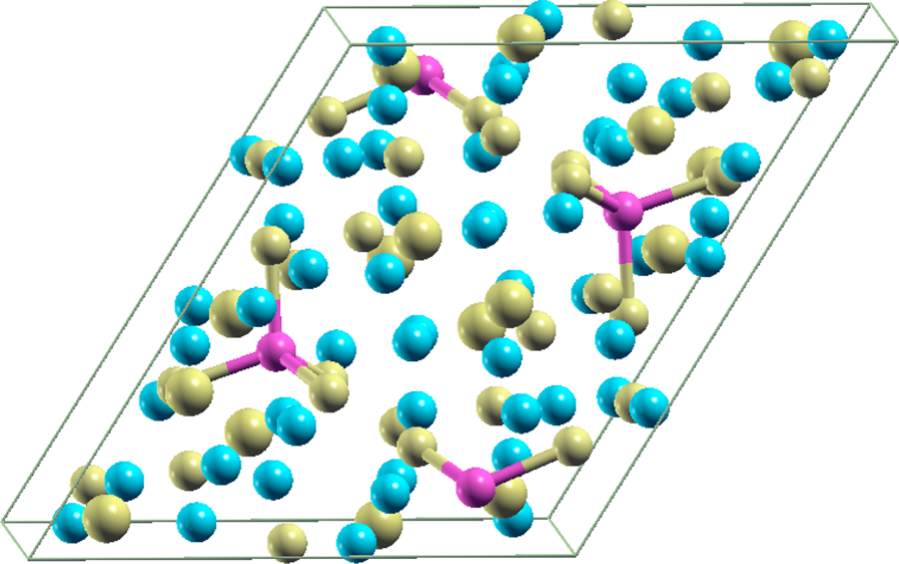
Primary unit cell of Yb_14_MgSb_11_; pink color is for Mg, cyan for Yb, and yellow for Sb.

**Figure 2:**
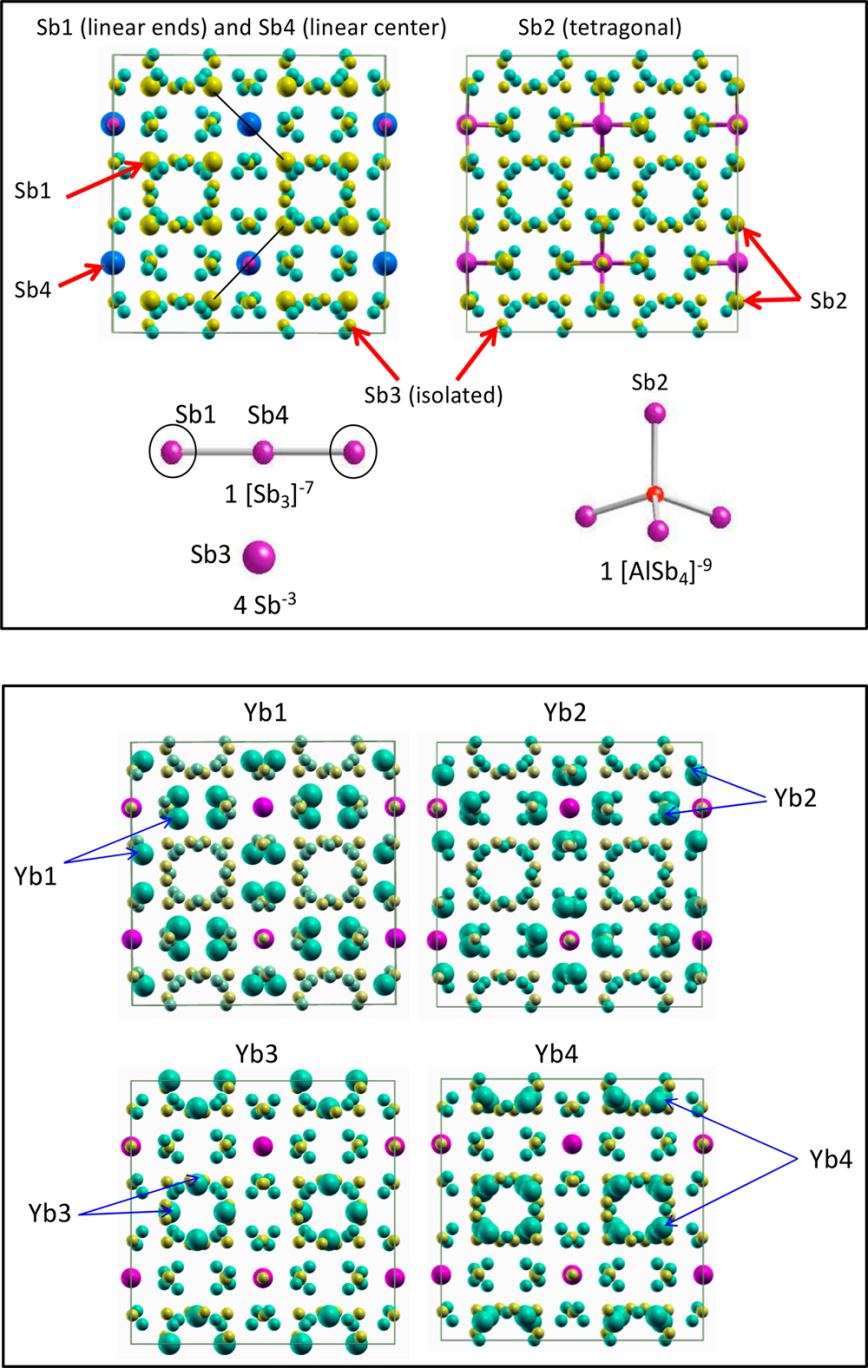
Conventional cells plotted at different views to show four different Sb sites (upper panel) and four different Yb sites (lower panel). The diameters of the Sb and Yb atoms at sites 1–4 are magnified for better visibility.

Yb_14_MgSb_11_ is isostructural with Ca_14_AlSb_11_ and crystallizes in the tetragonal space group *I*41/*acd* (see Figure 1). In this crystal structure, there are four inequivalent Sb sites with Wyckoff positions Sb1(16f), Sb2(32g), Sb3(32g), and Sb4(8b). Figure 2 shows the conventional unit cell viewed along the *c*-axis, illustrating the four Sb positions. The substitution of Sb atoms by As atoms is thus also classified into four groups corresponding to the four different positions of Sb. We labeled the substitutions at the Sb1, Sb2, Sb3, and Sb4 sites as groups 1, 2, 3, and 4, respectively (see Figure 2). Chemically, group 1 corresponds to the substitution of all Sb1 atoms at the two terminals of the linear chain [Sb_3_]^−7^ with As atoms. Group 2 denotes the substitutions of all Sb atoms at the corners of a tetrahedron [MgSb_4_]^−9^. Group 3 stands for the substitutions at the isolated ions Sb^–3^. Group 4 indicates the substitution at the center Sb atom of the linear chain [Sb_3_]^−7^. Groups 0 and 5 label Yb_14_MgSb_11_ (i.e., no substitution) and Yb_14_MgAs_11_, respectively.

The atomic basis vectors of all structures were relaxed for various fixed lattice parameters. The onsite correction (*U*) is applied to the 4f orbitals of the Yb atoms and tuned in order to obtain a good agreement with the experimental values of both the lattice constant and the pseudogap (*E*_g_) of Yb_14_MgSb_11_ (Yb_14_MgSb_11_ is weakly metallic). Since there is no experimental data available for Yb_14_MgSb_11–*x*_As_*x*_, as a first order of approximation, the so-determined value of *U* for Yb_14_MgSb_11_ is used for calculating the structural and electronic properties of Yb_14_MgSb_11–*x*_As_*x*_. We found that the lattice constant and the gap increase when increasing the value of *U*. For *U* = 0, the 4f peak is located in the gap, and Yb_14_MgSb_11_ is strongly metallic. As *U* increases, the 4f peak moves into the valence band. At the same time, we observe that the lattice constant also increases with *U*. Therefore, *U* is chosen to obtain a good agreement with the measured values of both the lattice constant and the pseudogap. (For convenience, we will label the term pseudogap as the “band gap” in further discussions.) In this work, the value of *U* is chosen to be 12 eV.

## Results and Discusion

### Electronic Properties

For Yb_14_MgSb_11_, the computed lattice constants were found to be *a* = 16.786 Å and *c* = 22.541 Å, in good agreement with the experimental values of 16.625 Å and *c* = 22.325 with an error of about 0.97% and ∼1% in *a* and *c*, respectively.

**Figure 3:**
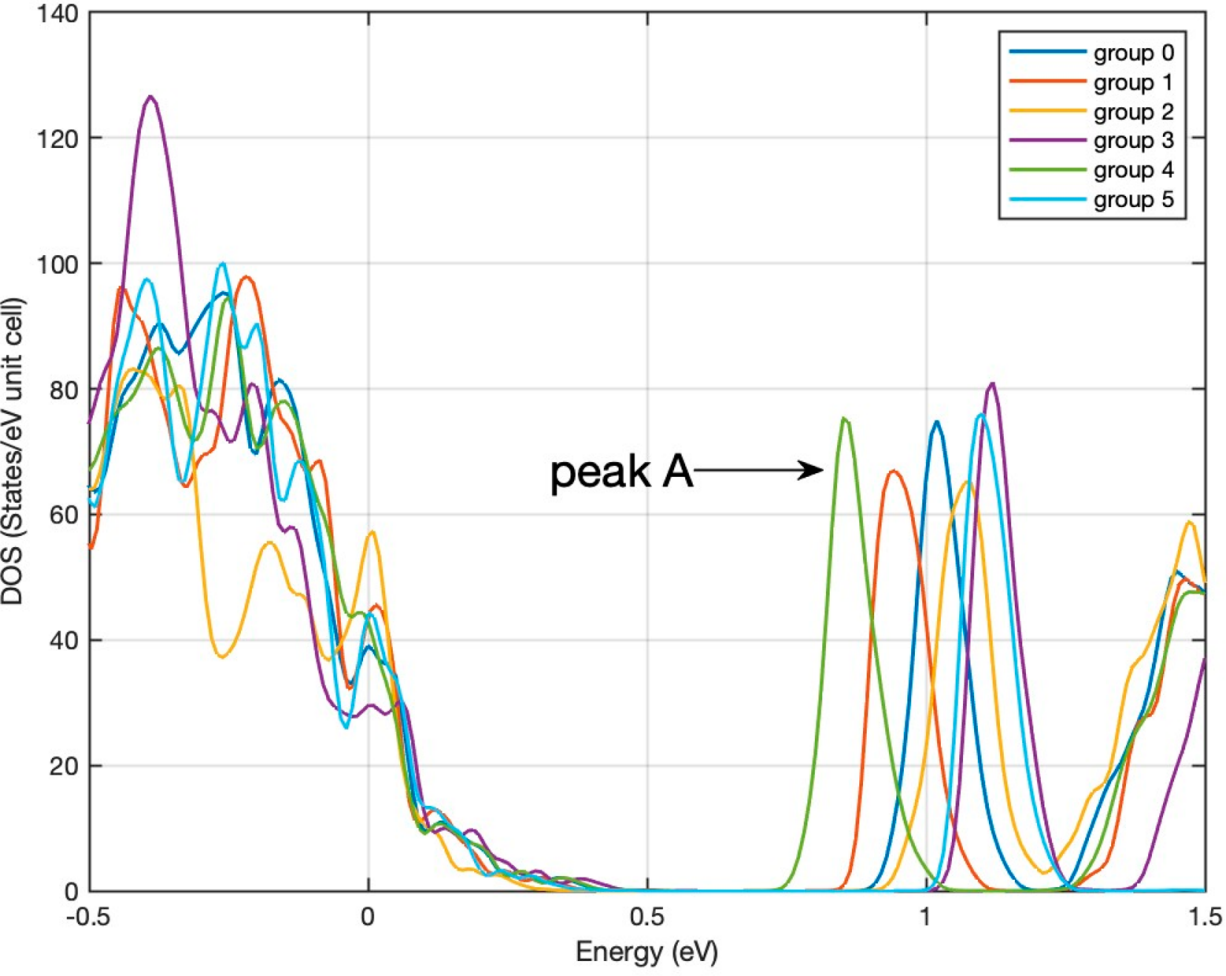
DOS of Yb_14_MgSb1_1–*x*_As_*x*_ (*x* = 0–1) with different groups.

Figure 3 shows the density of states (DOS) for Yb_14_MgSb_11–*x*_As_*x*_ (groups 1–4), Yb_14_MgSb_11_ (group 0), and Yb_14_MgAs_11_ (group 5). As the content of As atoms varies, the DOS changes significantly. Depending on the substitution site, the band gap can become smaller or larger than that of Yb_14_MgSb_11_ due to the shift of the first peak (peak A) at the band edge in the conduction band CB to either lower or higher energies, respectively. Table 1 lists the band gap and number of Sb atoms being substituted by As atoms for each group. The band gap is the largest for Yb_14_MgAs_11_, followed by groups 2, 3, 0, 1, and 4. Compared to the base compound Yb_14_MgSb_11_, while the gap increases for groups 5, 2, and 3, it decreases for groups 1 and 4. We also observe that the computed band gap for Yb_14_MgSb_11_ is smaller than the “expected” experimental gap (∼0.58–0.7 eV).^30,31^ For thermoelectric materials, the experimental value of the band gap *E*_g_ is often estimated by the Goldsmid–Sharp formula^41^*E*_g_ = 2*eTS*_max_, where *S*_max_ is the maximum Seebeck coefficient, *T* is the temperature corresponding to *S*_max_, and *e* is the electron charge. The experimental band gap covers a wide range of values. The band gap from the work by Hu et al. is extrapolated to be 0.7 eV, but that reported by Perez et al. is 0.58 eV.^31^ The computed band gap is smaller than the experimental value due to the well-known shortcoming of DFT, which underestimates the energies of empty states. Even though onsite correction was applied to Yb in our computation, an error of 17–31% in the gap is observed. Without applying onsite correction on Yb(4f), the gap is 0 due to a large peak of the 4f orbitals located in the gap, causing instability and difficulties in the numerical convergence. Larger values of the band gap can be obtained by applying an onsite correction for the Sb atoms. For example, by choosing *U*(Sb) = 2.0 eV, we obtain a band gap of ∼0.59 eV. The application of an onsite correction for Sb affects the band edge of the valence band, increasing the band gap because the main contribution to the valence band edge comes from Sb states. Without the onsite correction, the bonding with the Sb atoms is over-delocalized, therefore reducing the gap. Since there is no experimental data reported for Yb_14_MgSb_11–*x*_As_*x*_ compounds that would allow us to tune *U*(Sb) and *U*(As), the onsite correction is only applied on Yb(4f). This approximation is sufficient for the scope of this study, which is to understand the chemical activity of the different Sb sites, and the relative trends and effects of the site substitutions on the electronic and transport properties.

**Table 1:** The Pseudogap of Yb_14_MgSb_11–*x*_As_*x*_

Group	Number of Sb substituted	Gap (eV)
Yb_14_MgSb_11_	0	0.482
Group 1	8	0.476
Group 2	16	0.629
Group 3	16	0.571
Group 4	4	0.318
Yb_14_MgAs_11_	44	0.635

**Figure 4:**
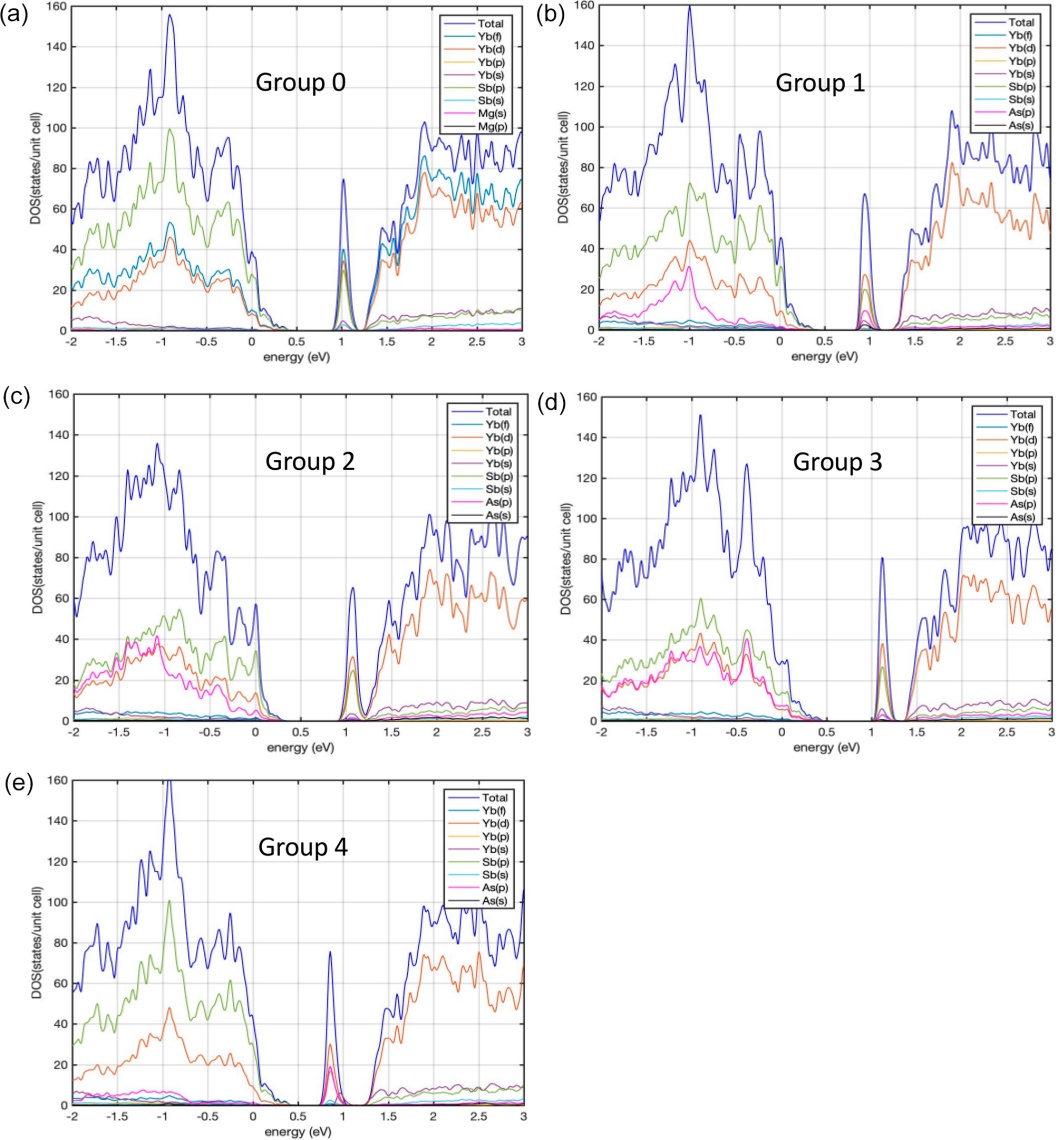
Partial DOS (PDOS) of groups 0–4 labeled as Group 0 to Group 4, respectively. The DOS for all orbitals of the same atom are summed up.

The impact of the various site substitutions on the DOS can be further understood by investigating the partial DOS (PDOS). Figure 4 shows the PDOS for Yb_14_MgSb_11_, groups 1–4, and Yb_14_MgAs_11_. Table 2 gives the atomic orbital analysis of the HOMO and LUMO states for the different groups. We found that within the range of energy important for transport properties all of the groups share the following common features: in the valence band (VB), the Sb atoms have the largest contribution to the total DOS, followed by Yb atoms. The band edge of the VB is dominated by the Sb (5p) atomic orbitals. The 4f from Yb becomes dominant only for energies larger than 1 eV from the valence band edge. In contrast, the largest contribution in the CB comes from Yb atoms (mainly Yb(4f) and Yb(5d)) followed by Sb(5p). The contribution from Mg atoms is negligible compared to other atom types in both the CB and VB.

**Table 2:** Contribution from the As Atoms to the HOMO and LUMO States for the Different Groups

HOMO	Group 1 (8 As)	Group 2 (16 As)	Group 3 (16 atoms)	Group 4 (4 As)
1	0.064 (p)^a^	0.176 (p)	0.256 (p)	0
2	0.008 (p)	0	0.208 (p)	0.052 (p)
3	0	0.112 (p)	0.128 (p)	0.064 (p)
4	0.032 (p)	0.048 (p)	0.128 (p)	0.064 (p)
5	0.032 (p)	0.048 (p)	0.160 (p)	0

LUMO	Group 1 (8 As)	Group 2 (16 As)	Group 3 (16 atoms)	Group 4 (4 As)
1	0.144 (p)	0	0	0.256 (p)
2	0.144 (p)	0	0	0.256 (p)
3	0.128 (p)	0.016 (p)	0.048 (p)	0.208 (p)
4	0.128 (p)	0.016 (p)	0.048 (p)	0.208 (p)
5	0	0.048 (p)	0.112 (p)	0.008 (p)

a The letter p in parentheses denotes the 4p orbital.

Since the bonding nature and electronic structure in the vicinity of the band edge of semiconducting material mostly determine the transport properties, analyzing and comparing the PDOS of different groups near the band edge helps for understanding the effect of substitution of each Sb site by As. Figure 4 shows that within the energy range significant for transport properties the contribution to the DOS of the CB and VB varies from one group to another. The contribution from As atoms to the first sharp peak (denoted as peak A, see Figure 3) in the CB is the largest for group 4, followed by groups 1, 3, and 2 (see Figure 4b–e). Although having a smaller number of As atoms per unit cell (8 As atoms for group 1 and 4 As atoms for group 4), groups 1 and 4 still have larger As contributions to peak A than groups 2 and 3. This means that the Sb4 and Sb1 sites play a larger role in forming peak A than other sites. In contrast, a much larger As contribution (mainly by 4p orbitals) to the DOS in the VB comes from groups 2 and 3. Comparison of groups 2 and 3, both of which have the same number of As atoms per unit cell, shows that the slope of the DOS near the band edge is steeper for group 2 than for group 3, and this can affect transport properties, which will be discussed in more detail in the next section.

The differences observed in the band gap and the contributions to the band edges in the VB and CB can be understood by analyzing the atomic orbital contributions to the first four bands in the VB and the CB for each group (see Table 2). It is known that As atoms have lower energy (i.e., atomic and orbital energy) but larger electronegativity than Sb atoms. Replacing Sb atoms with As is expected to change local polarization, bond length, and band energy. Table 2 shows the contribution of atomic orbitals (AOs) to the first four bands in the CB and VB. Among all of the groups, the contribution from the As atoms to the LUMO is the largest for group 4, followed by group 1, and then groups 2 and 3. Since for group 4 the substitution occurs at the center of the linear chain [Sb_3_]^−7^, replacing the central Sb atom (Sb4 site) with an As atom of larger electronegativity and lower energy causes the orbital overlap between atoms in the linear chain to become weaker, and the bonds become more polarized. Consequently, the energy gap between the bonding and anti-bonding states decreases, leading to the shift of the LUMO to lower energy. Because the contribution from As atoms to the LUMO for group 4 is the largest among all groups, the shift of the LUMO is the largest. In addition, the HOMO of group 4 has no contribution from the As atoms, whereas the contributions from other atoms to the HOMO remains unchanged compared to group 0. Therefore, the HOMO is almost not shifted, while the LUMO is significantly shifted, causing the largest reduction in the band gap for group 4.

A similar analysis can be applied to group 1, where two Sb atoms at the terminal positions in the linear chain (site Sb1) are replaced with two As atoms. Replacing Sb atoms with As atoms at the Sb1 sites also leads to a decrease in the gap between bonding and anti-bonding but to a lesser degree compared to group 4 (Sb4 sites) because the contribution from the As atoms to the LUMO is less than that for group 4 (see Table 2). For the HOMO, compared to group 4, there is a larger contribution from As atoms to the HOMO. However, we do not see a shift to higher energy caused by the bond weakening. This is because the position of a band energy is determined by a net effect of different factors including the difference in the system energy due to having different numbers of As atoms in the system, the degree of the band energy shift due to bond weakening, and the relative changes in the contributions of the atoms, compared to the reference group (group 0). While the first factor tends to shift the whole system energy to lower values, the second factor tends to shift the HOMO to higher energies for the cases of groups 1 and 4. Since for group 1 the system has more As atoms than that for group 4, the observed net effect is that the valence band edge is at lower energy than for group 4. Therefore, group 1 has a larger band gap compared to group 4.

While the band gap is shrinking for groups 1 and 4, it becomes larger for group 2. In contrast to groups 1 and 4, where the substitution happens on the same linear chain, for group 2, the substitution occurs at the corners of the tetrahedrons of [MgSb_4_]^−9^. Replacing the Sb atoms with As having lower energy and larger electronegativity will further strengthen the bonds because the differences in diameter and energy between As and Mg atoms are smaller, resulting in stronger overlap between Mg and As orbitals. This causes the LUMO to shift further to higher energy and the HOMO to lower energy. Therefore, the gap between bonding and anti-bonding is expected to expand compared to groups 1 and 4. The larger increase in the gap for group 2 can also be explained quantitatively using the AO analysis in Table 2. While the contributions of the As atoms to the LUMO in the cases of groups 1 and 4 are large, it vanishes for group 2 (see Table 2).

For group 3, since the substitution occurs at the isolated Sb3 sites, the bonding with the Yb atoms is mainly ionic because these atoms play the role of ensuring charge neutrality. Replacing Sb atoms with As having larger electronegativity tends to increase the charge transfer and strengthen ionic bonds between As and Yb atoms. Therefore, we expect to see an increase in the gap between the HOMO and LUMO. We found that the energy level of the HOMO is the lowest for group 2 and the highest for group 3. Besides the factors discussed above, group 2 has the largest increase (double or more) in the contribution of Yb(5d) orbitals to the HOMO. This means that more empty Yb(5d) orbitals participate in the bonding for group 2 and thus further stabilize the HOMO and lower the HOMO energy compared to other cases. On the other hand, we only observe a slight decrease in the Yb(5d) orbital contribution for other groups (1, 3, and 4). This therefore explains why the HOMO for group 3 is higher than that for group 2 and the increase in the band gap for group 3 is smaller than that for group 2.

**Figure 5:**
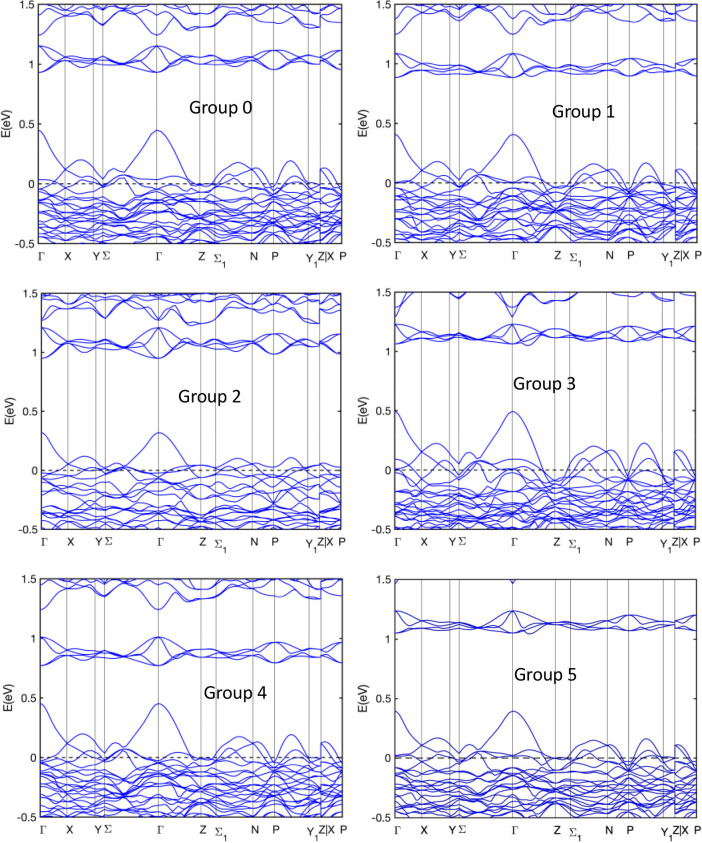
Band structure of Yb_14_MgSb_11–*x*_As_*x*_ (groups 0–5). The labels Group 0 to Group 5 denote group 0 to group 5, respectively.

In order to gain further insight into the effect of substitution on the electronic and transport properties, the band structures for the different cases are also compared (see Figure 5). By comparing all band structures, we observe that the DOS first peak in the CB is identified with a small group of four to five bands in the CB, and the conduction band edge change is sensitive to the substitution sites. The magnitude of the band gap is determined by the shifting of these bands upon the substitution of Sb by As atoms. For all cases, the band gap is direct. While at the gamma point the top of the VB exhibits a sharp valley, the curvature of the first band in the CB is much flatter. In other words, the top valence band is a light band, whereas the lowest conduction band is a heavy band. The curvature of the first CB and the first VB and the band offset (defined as the energy difference from the top of the VB to the Fermi level at 0 eV) are found to vary from one group to another. Table 3 presents the computed effective mass for Yb_14_MgSb_11–*x*_As_*x*_ at gamma. The mean effective mass is computed in two different ways: the geometric mean and the harmonic mean. The geometric mean hole effective mass is defined as , where *g* is the number of valleys. In this case, *g* = 1. The harmonic mean hole effective mass is calculated as . All of the cases share the common feature of having an anisotropic effective mass. For the VB, *m*_*x*_ = *m*_*y*_ ≠ *m*_*z*_, but for the CB, *m*_*y*_ = *m*_*z*_ ≠ *m*_*x*_. The anisotropy in the effective mass is smaller for hole carriers than for electron carriers. In addition, the effective masses are much larger for the electrons than for the holes. The geometric average of the effective mass of the top valence band *m*_pg_ is the largest for group 2 (0.31 *m*_e_) and the smallest for group 3 (0.24 *m*_e_). For the lowest conduction band, the effective mass *m*_ng_ is the largest for group 3 (2.51 *m*_e_) and the smallest for group 2 (1.13 *m*_e_). The ratio of *m*_ng_/*m*_pg_ varies from 3.88 to 10.59, where the largest value corresponds to group 3 and the smallest value corresponds to group 2. The variation in the effective mass can result in differences in the electrical conductivity σ, since, in simple transport models, σ is inversely proportional to the effective mass. The harmonic mean effective masses give a similar trend as the geometric mean.

**Table 3:** Effective Mass at Gamma for Yb_14_MgSb_11–*x*_As_*x*_

Compound	*m*_*x*_	*m*_*y*_	*m*_*z*_	*m*_pg_^a^	*m*_ph_^a^	*m*_*x*_	*m*_*y*_	*m*_*z*_	*m*_ng_^b^	*m*_nh_^b^	*m*_ng_/*m*_pg_
Group 0	0.20	0.20	0.44	0.26	0.24	1.9	1.08	1.08	1.30	1.26	5.01
Group 1	0.21	0.21	0.48	0.28	0.26	2.4	1.30	1.30	1.59	1.53	5.77
Group 2	0.23	0.23	0.59	0.31	0.29	2.25	0.90	0.90	1.22	1.13	3.88
Group 3	0.18	0.18	0.41	0.24	0.22	3.09	2.26	2.26	2.51	2.48	10.59
Group 4	0.20	0.2	0.42	0.26	0.24	1.66	1.21	1.21	1.34	1.33	5.25
Group 5	0.22	0.22	0.57	0.30	0.28	2.85	2.15	2.15	2.36	2.34	7.82

a *m*_pg_ and *m*_ng_ denote the geometric mean effective masses for the valence and conduction bands, respectively.

b *m*_ph_ and *m*_nh_ denote the harmonic mean effective masses for the valence and conduction bands, respectively.

### Transport Properties

**Figure 6:**
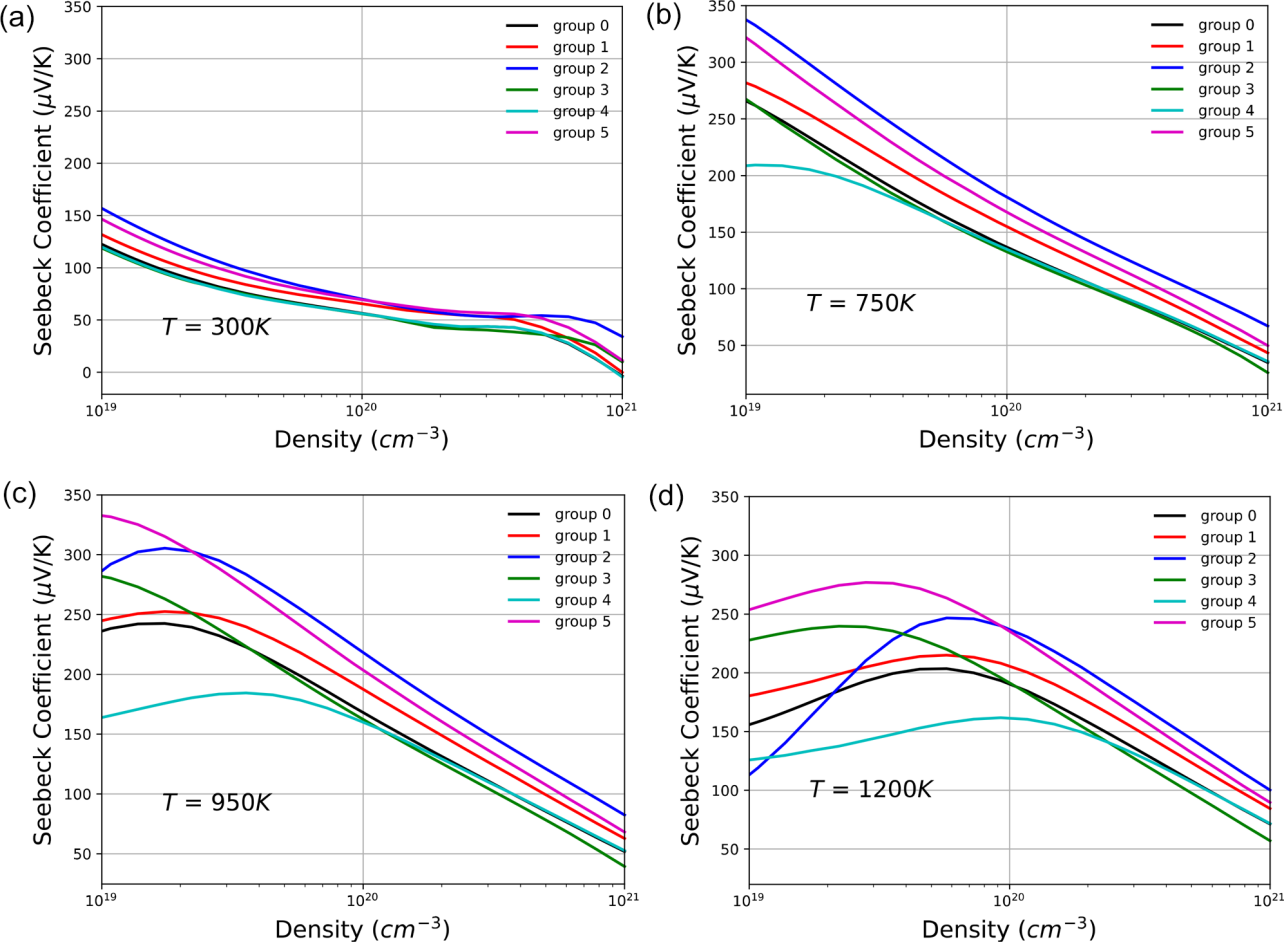
Seebeck coefficients for Yb_14_MgSb_11–*x*_As_*x*_ as a function of density for various temperatures *T* (300, 750, 950, and 1200 K).

**Figure 7:**
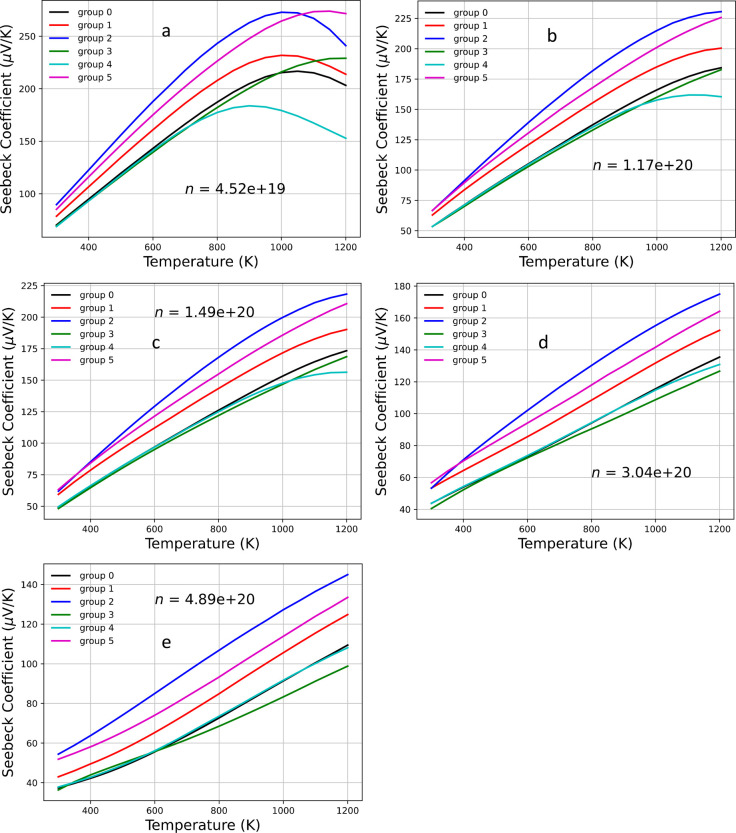
Seebeck coefficients for Yb_14_MgSb_11–*x*_As_*x*_ as a function of temperature *T* for various carrier concentrations: (a) *n* = 4.52 × 10^19^, (b) *n* = 1.17 × 10^20^, (c) *n* = 1.49 × 10^20^, (d) *n* = 3.04 × 10^20^, and (e) *n* = 4.89 × 10^20^.

The Seebeck coefficient and electrical conductivity for Yb_14_MgSb_11–*x*_As_*x*_ are computed using standard expressions derived from the linearized Boltzmann transport equation (BTE)^42−45^ and the rigid band approximation to describe the variation of the electron/hole concentration due to the vacancies.^46−48^ In the rigid band approximation, the band structure is assumed to be unchanged when varying the carrier concentration; only the position of the Fermi energy is varied. We assume that the scattering process is mainly caused by acoustic phonons, and the relaxation time is approximated as , where *E* is the energy and τ is the relaxation time. The prefactor τ_0_ is chosen to be 8 × 10^–14^ s and was obtained by fitting the computed Seebeck coefficient and electrical resistivity to the measured data reported by Perez et al.^31^Figure 6 presents the plots of the Seebeck coefficient as a function of concentration for Yb_14_MgSb_11–*x*_As_*x*_, where *x* varies from 0 to 1 for temperatures of 300, 750, 950, and 1200 K. Figure 7 illustrates the plots of the Seebeck coefficient as a function of temperature. In general, the Seebeck coefficient increases with increasing temperature, reaching a maximum and then decreasing at larger temperatures. The decrease in the Seebeck coefficient with increasing density is due to the increase in the contribution from the electronic bands to the Seebeck coefficient for a p-type material. Except for groups 3 and 4, for temperatures from 300 to 1200 K, the Seebeck coefficient for all other groups increases compared to the base compound, Yb_14_MgSb_11_. As the temperature increases, the difference in the Seebeck coefficient for different groups becomes larger. This trend results from the combination of different factors including changes in the effective mass (*m*_p_^*^), band gap (*E*_g_), fraction or number of bands contributing to the Seebeck coefficient, and electron scattering. The effects of these factors on the Seebeck coefficient manifest themselves in complicated ways. First, from the single band approximation, the relationship between the Seebeck coefficient *S* and *m*_p_^*^ can be expressed as^46^
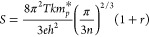
2where *r* is the scattering factor, *k* is the Boltzmann constant, *m*_p_^*^ is the effective mass, and ℏ is the Plank constant. Equation 2 shows that *S* increases with *m*_p_^*^. Second, *S* decreases with a decreasing band gap due to an increasing contribution from the electrons for a p-type compound. Therefore, having a large *m*_p_^*^ but small band gap can lead to a cancelation effect, and the net effect depends on which factor dominates in a given range of *T* and carrier concentration *n*. In addition, the third factor, the band contribution, also plays a very important role in determining the value of *S*. A larger fraction of bands contributing to the Seebeck coefficient can increase *S*. Finally, electron scattering also affects the Seebeck coefficient in different ways (increasing and decreasing), an effect that is not fully reflected in the constant relaxation time approximation (CTR). In other words, all of these factors can either have additive or canceling effects on the value of the Seebeck coefficient.

**Figure 8:**
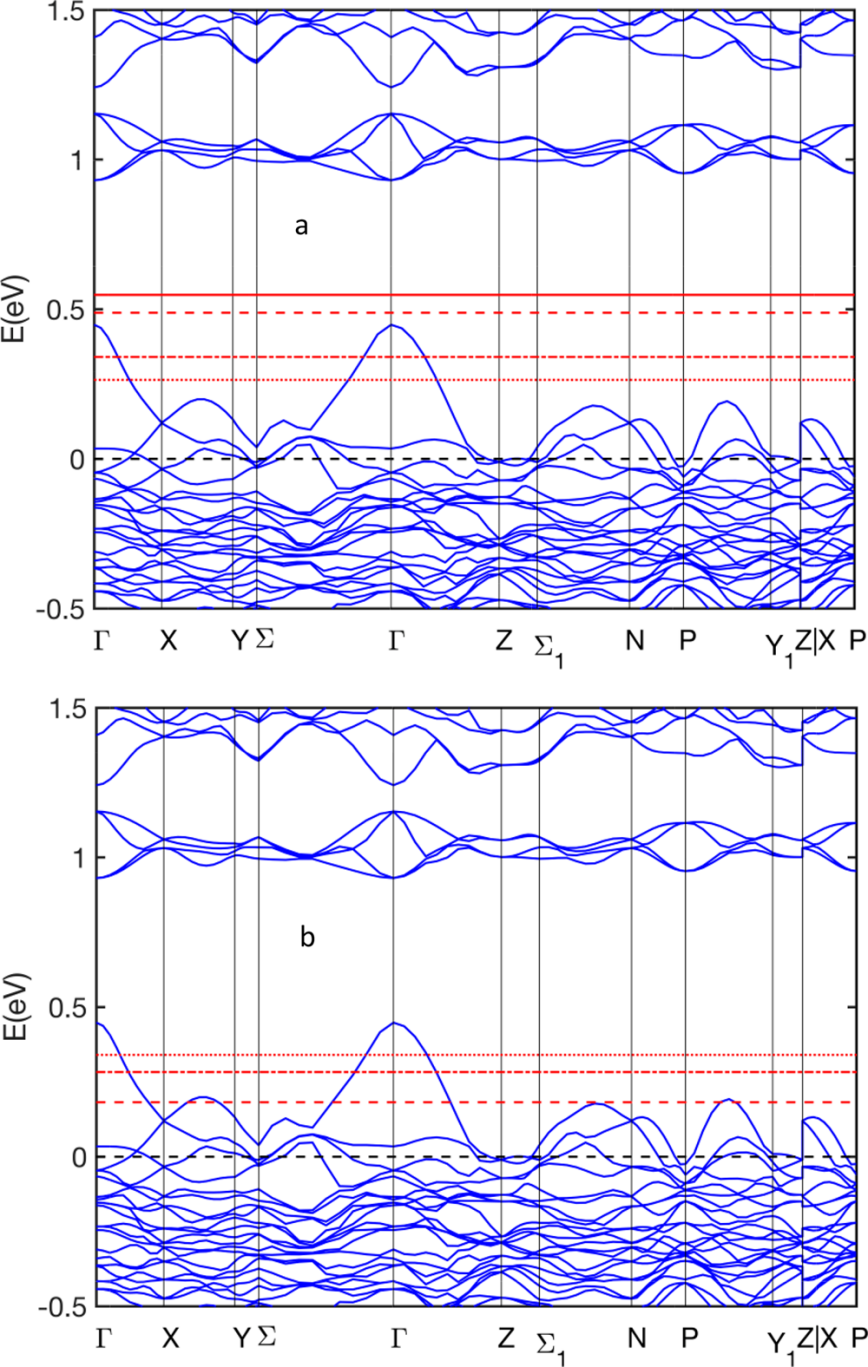
(a) Changes in chemical potential with carrier density for Yb_14_MgSb_11_ as an example: the solid red line is for *n* = 4.89 × 10^18^, the dashed line is for *n* = 3.29 × 10^19^, the dash-dotted line is for *n* = 1.61 × 10^20^, and the dotted line is for *n* = 3.04 × 10^20^. (b) Changes in the chemical potential with temperature for Yb_14_MgSb_11_ as an example; *n* = 1.61 × 10^20^; the dashed line is for *T* = 300 K, the dash-dotted line is for *T* = 950 K, and the dotted line is for *T* = 1200 K.

**Figure 9:**
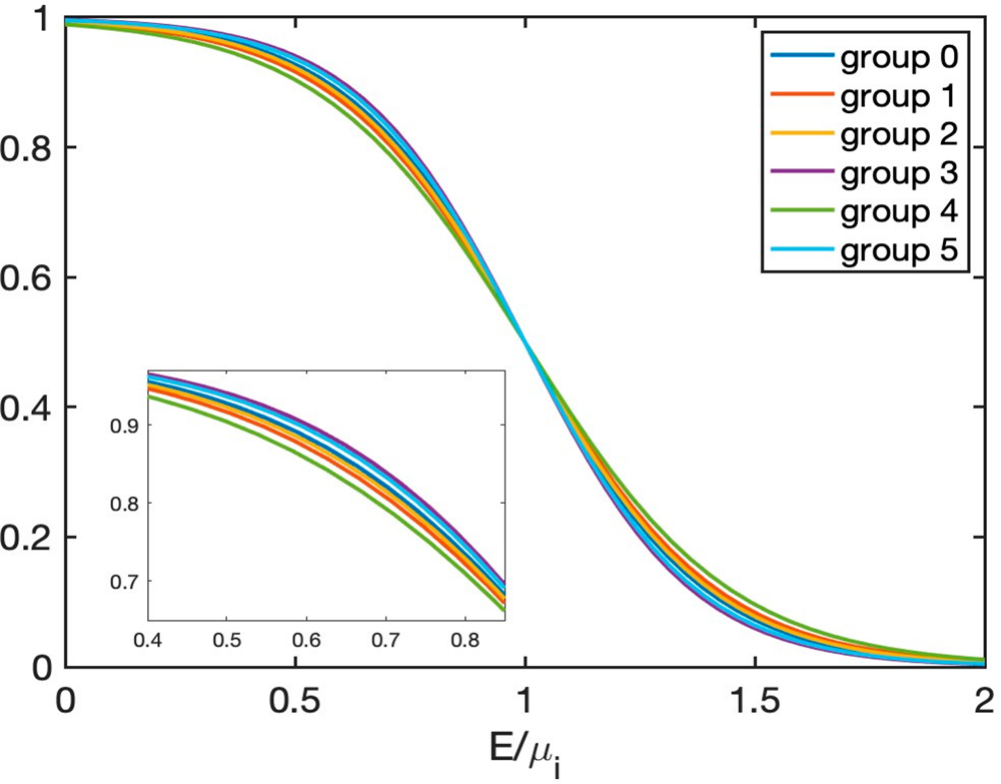
Fermi Dirac distribution function for different groups plotted for *T* = 1200 K. *E* denotes the energy; *μ*_*i*_ denotes the chemical potential of group *i*. The inset shows a subregion of the plot with higher resolution.

In order to estimate how many bands contribute to the Seebeck coefficient, we plotted the chemical potential μ as a function of *n* and the Fermi Dirac distribution function for each group (for an example, see Figures 8 and 9). The former determines the location of the chemical potential corresponding to a given carrier density. The latter shows how many bands are occupied as a function of temperature. From Figure 8, the higher the carrier density, the lower the value of μ, and the larger the fraction of band(s) contributing to the Seebeck coefficient. Furthermore, the higher the temperature, the closer the value of μ to the CB minimum (CBM), the larger the electron contribution to the Seebeck coefficient, and the smaller the fraction of VB bands contributing to *S*. In addition, at higher *T*, the Fermi distribution function is smeared further, and a larger fraction of band(s) is occupied. Combining all of this information with the location of the valence and conduction band edges (VBM and CBM), we can evaluate the relative electron contributions for different groups to the Seebeck coefficient. Based on this analysis together with the *m*_p_^*^ and band gap, we found that, for high carrier density *n* and high temperature, e.g., for *n* ≥ 3.04 × 10^20^ and *T* > 900 K (see Figure 7d,e and Table 3), the effective mass is the determining factor in explaining the trend in *S*. In these cases, the value of *S* is seen to increase with increasing *m*_p_^*^. Even though the difference in *S* is smaller for groups 0, 3, and 4 due to a smaller difference in *m*_p_^*^ that can cause other factors such as band contribution, band gap, and electron contribution to become more competitive, the final results still follow the trend predicted by eq 2. The reason is that, at high *n* and *T*, while higher *T* causes μ to increase, higher *n* results in a decrease in μ value (see Figure 8). Due to the near canceling effect of increasing and decreasing μ at higher *n* and *T*, *m*_p_^*^ becomes the determining factor. On the other hand, as *n* increases and *T* decreases, both have an additive effect on μ, reducing μ. In this case, the fraction of band(s) contributing to *S* increases, while the contribution from the electrons decreases. At low enough *T*, the effects of a larger band contribution and a smaller electron contribution to *S* become increasingly more important and compete with the effective mass factor. Therefore, *m*_p_^*^ is no longer the only factor to determine *S*. For instance, for *T* < 600 K and *n* = 4.89 × 10^20^ (see Figure 7e), we start to see a crossover in the value of *S* even though group 3 has a smaller *m*_p_^*^ than groups 0 and 4.

Similarly, at low *n* and high *T*, e.g., *n* < 1 × and *T* > 940 K (see Figure 6c, d and Figure 7a), since both higher *T* and lower *n* cause μ to increase (additive effect), the band contribution decreases, but the contribution from the electrons becomes more important. For this range of temperature and density, all three factors become competing factors, where the contribution from the electrons becomes more pronounced than that of high *T* and *n*. We also note that, for group 2, as *n* decreases passing the point *n* ∼ 5 × 10^19^, the Seebeck coefficient bends over and decreases much faster than for other groups. The fast decrease results from the faster reduction in band contribution because group 2 has the lowest VB minimum (VBM). We observe that, for *n* ≥ 1.61 × 10^20^, the value of μ starts to be higher than the VBM, while μ is still smaller than the VBM for other groups. It is noted that, even though μ is higher than the VBM, a fraction of the band(s) is still occupied due to thermal excitation, as determined by the Fermi Dirac distribution function for a given *T*.

**Figure 10:**
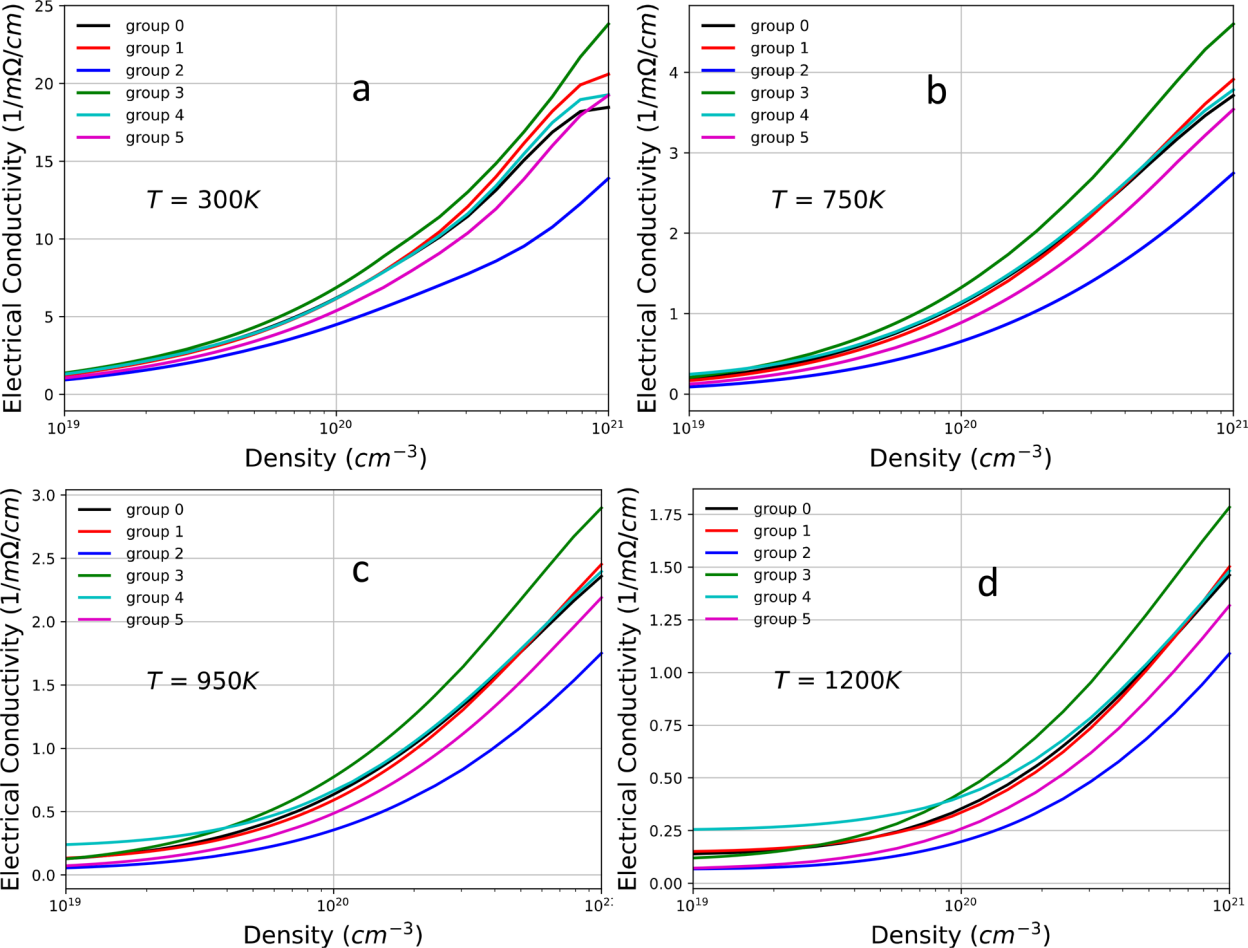
Electrical conductivity as a function of carrier concentration for various temperatures: (a) *T* = 300 K, (b) *T* = 750 K, (c) *T* = 950 K, and (d) *T* = 1200 K.

**Figure 11:**
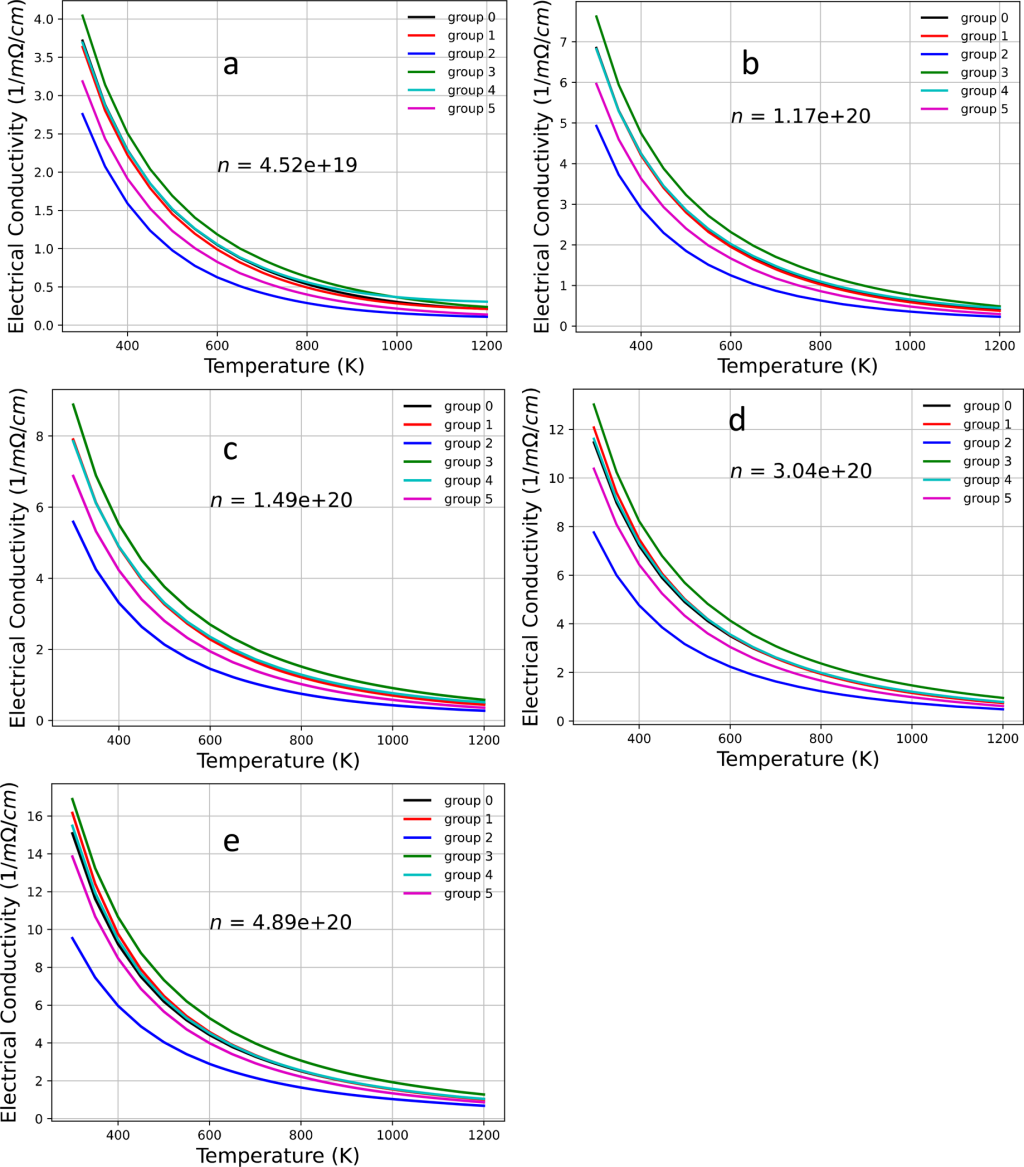
Electrical conductivity as a function of temperature *T* at various carrier concentrations: (a) *n* = 4.52 × 10^19^, (b) *n* = 1.17 × 10^20^, (c) *n* = 1.49 × 10^20^, (d) *n* = 3.04 × 10^20^, and (e) *n* = 4.89 × 10^20^.

Figures 10 and 11 show the plots of the electrical conductivity σ. Except for groups 2 and 3, the changes in σ are small for most of the groups when compared to the base compound, Yb_14_MgSb_11_. In general, the trend in electrical conductivity is consistent with the change in the effective mass compared to the base compound. The decrease and increase in σ for groups 2 and 3, respectively, are significant. However, while the dramatic change in σ for group 2 seems to be consistent with the larger change in the value of effective mass *m*_p_^*^ (see Table 3), for group 3, the larger increase of the σ value cannot be attributed directly and only to the small decrease in *m*_p_^*^ compared to that of the base compound. This large deviation probably originates from differences in the details of the DOS near the band edge such as the slope of the DOS and other factors, as previously discussed for the Seebeck coefficient.

**Figure 12:**
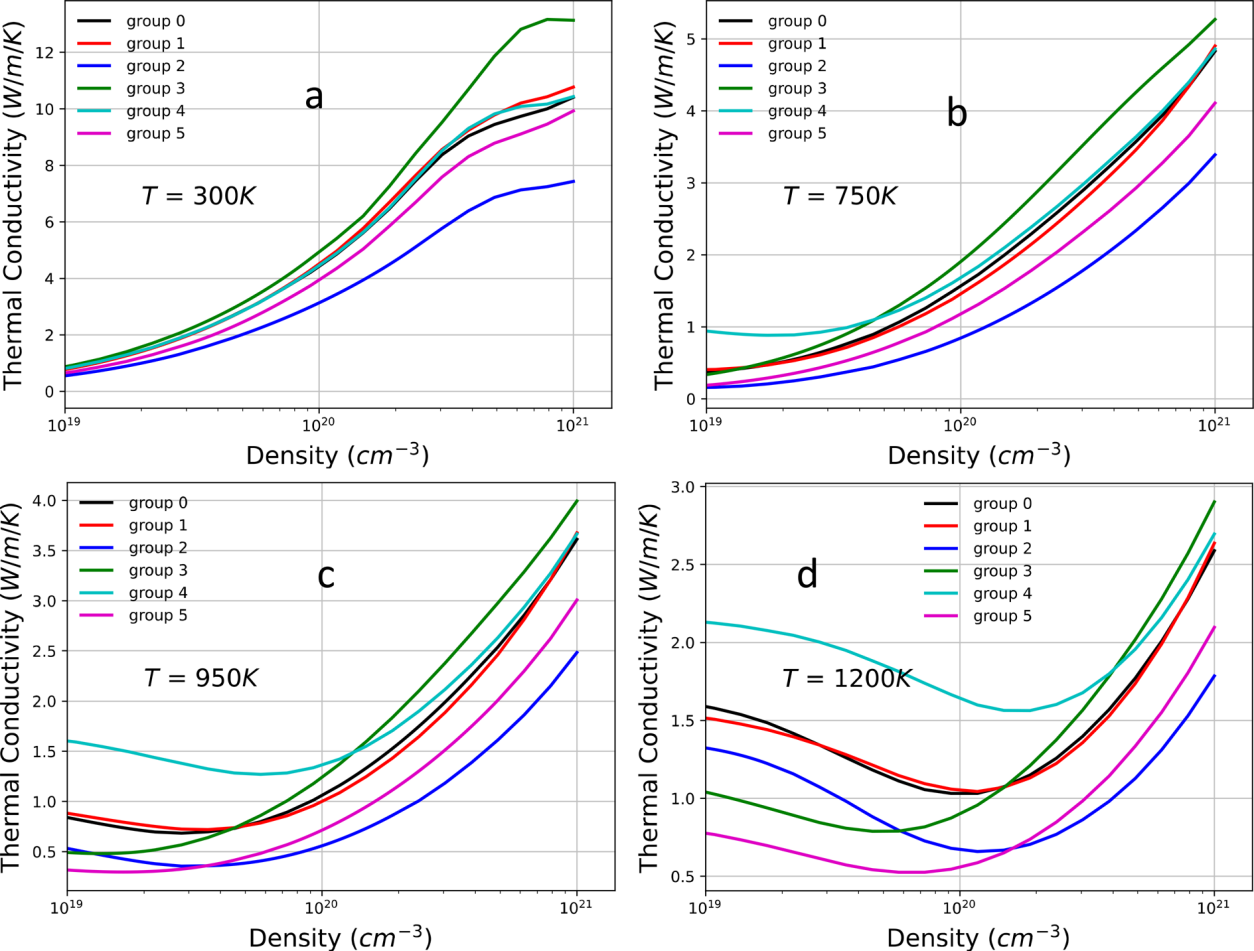
Electronic thermal conductivity as a function of carrier density for various temperatures: (a) *T* = 300 K, (b) *T* = 750 K, (c) *T* = 950 K, (d) *T* = 1200 K.

**Figure 13:**
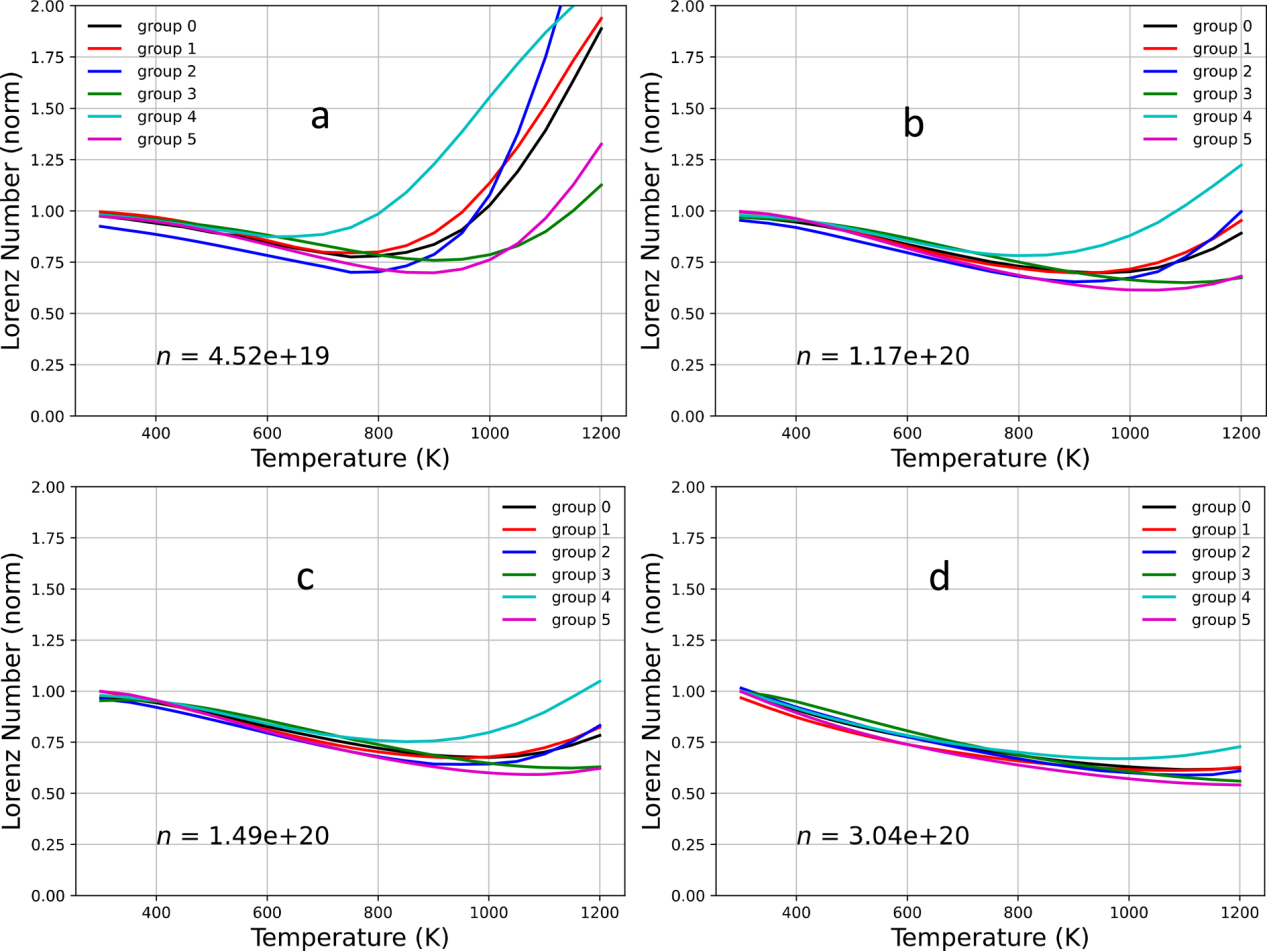
Normalized Lorentz number as a function of temperature *T* for various carrier concentrations: (a) *n* = 4.52 × 10^19^, (b) *n* = 1.17 × 10^20^, (c) *n* = 1.49 × 10^20^, and (d) *n* = 3.04 × 10^20^.

Similar to the electrical conductivity, we observe a large deviation in the electronic thermal conductivity *κ*_e_ (see Figure 12) for groups 3, 5, and 2 compared to group 0. While group 3 shows an increase in *κ*_e_, groups 2 and 5 have a decrease in *κ*_e_. This trend is consistent with the increase and decrease in electrical conductivity observed for groups 3, 5, and 2, respectively, following the Wiedemann–Franz law and the trends in the effective mass. However, as the temperature increases (*T* ≥ 950 K), and for *n* ≤ 1 × 10^20^, the differences in *κ*_e_ among the groups become larger and do not follow the trend in the effective mass anymore. We observe crossovers (larger *κ*_e_ for larger *m*_p_^*^). In order to illustrate this deviation, we plot the normalized Lorentz number *L* as a function of *T* (see Figure 13). In these plots, the Lorentz number is normalized to the theoretical value of 2.44 × 10^–8^ V^2^ K^–2^. We observe a large deviation from the value of 1 at high *T* and low density. In this range of *T* and *n*, the deviation is the largest for the group having the smallest band gap. As explained in the previous discussion for the Seebeck coefficient, increasing *T* and decreasing *n* causes the chemical potential to increase. The contribution of the electrons becomes more significant, and therefore, the group with a smaller band gap will be more sensitive to the change. Since group 4 has the smallest band gap, it has the largest change in *L* (see Figure 13a–c). However, at even higher *T* (*T* ≥ 1000 K) and lower *n* (*n* ≤ 1 × 10^20^), the value of *L* for group 2 dominates over the other groups, except for group 4. The reason is that, for the same value of μ, the fraction of band contribution to *L* for group 2 decreases faster than for the other groups because group 2 has a lower VBM than the other groups. This means that the contribution from the hole carriers drops faster, making the effect of the electron contribution become stronger. Consequently, the change in *L* for group 2 grows faster at higher *T*, and near 1200 K, *L* becomes larger than even group 4. A similar explanation applies to the cases of groups 5 and 3.

**Figure 14:**
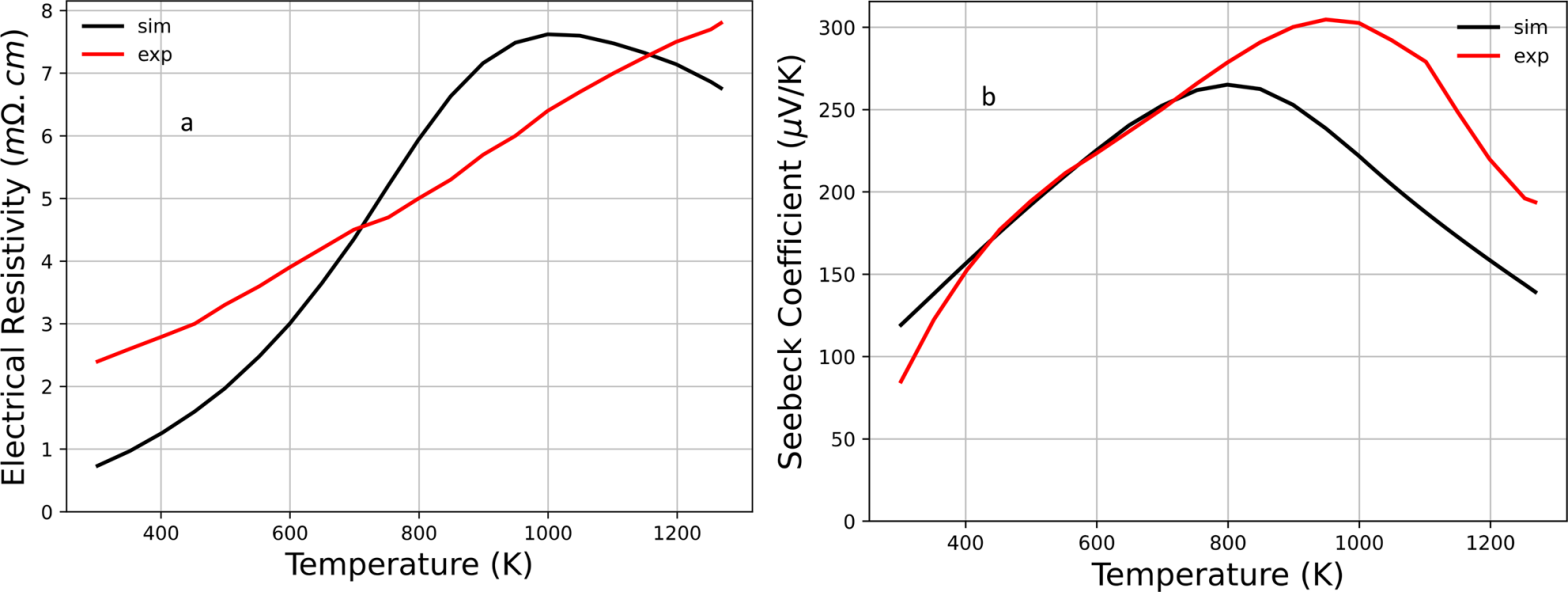
(a) Comparison of the experiment (red) and simulated (black) electrical resistivities. The red line represents the experimental data (labeled as “exp” in the legend).^31^ The simulation data is plotted in black color (labeled as “sim”). (b) Comparison of the experiment (red) and simulated (black) Seebeck coefficients. The same convention as in part a is used.

Finally, we compared the computed Seebeck coefficient for the base compound Yb_14_MgSb_11_ to the experimental values.^31^ We found the relative error ranges from 0 to 34%, depending on the temperature (see Figure 14). The largest deviation occurs at high temperatures (*T* > 750 K). The deviation can be attributed to a number of factors, including the relaxation time approximation, scattering mechanisms, the rigid band approximation, the fixed unit cell volume, and many-body interactions at high temperature. For example, at high temperature, the unit cell expands. Using a fixed size unit cell can cause errors in the calculations of the electron concentration due to changes in the band structure and volume. However, as we mentioned above, the main purpose of this work is to provide insight into the trends of how the thermoelectric properties change with selective atomic substitutions, and this is especially useful when experimental data is not yet available.

### Heat of Formation

We also computed the energy of formation for each group. The heat of formation can be calculated as^49^
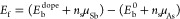
3where *E*_f_ is the heat of formation, *n*_s_ is the number of Sb atoms substituted by As, *E*_b_^dope^ and *E*_b_^0^ are the binding energies of a supercell with and without substitution, respectively, and *μ*_Sb_ and *μ*_As_ denote the chemical potentials for Sb and As.^50^

The binding energy is computed as the difference between the energy of the crystal structure (without substitution) and that of the isolated constituent elements (Yb, Sb, Mg, and As):
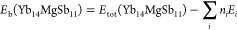
4Table 4 lists the binding energy and formation energy for the different groups. The formation energy is the largest (most negative) for group 5, followed by group 2 and group 3. Group 4 is the least stable compared to the other groups.

**Table 4:** Binding Energy and Formation Energy for Different Groups at 0 and 300 K

Group	ns	*E*_b_^a^ (eV)	*E*_f_^b^(0 K, eV)	*E*_f_(300 K, eV)	*E*_f_(0 K, kJ/mol)	*E*_f_(300 K, kJ/mol)
0	0	–304.55				
1	2	–307.97	–3.22	–3.48	–2.98	–3.23
2	4	–314.28	–9.27	–9.80	–8.60	–9.09
3	4	–312.33	–7.32	–7.85	–6.79	–7.28
4	1	–305.84	–1.22	–1.35	–1.13	–1.25
5	11	–332.32	–26.40	–27.84	–24.49	–25.83

a *E*_b_ denotes the binding energy.

b *E*_f_ is the formation energy, and ns is the number Sb atoms substituted by As ones per formula unit.

## Conclusions

In conclusion, we have used first-principles electronic structure calculations to systematically investigate the effect of selective atomic substitutions of Sb sites in Yb_14_MgSb_11_ with As atoms. We found that the substitution can result in significant changes in the electronic properties and thus the thermoelectric properties. Substitutions of Sb at the corner of the tetrahedron (group 2) are predicted to yield the largest increase in the Seebeck coefficient at high carrier concentration and high temperature. By providing insight into how selective atomic substitutions affect the chemical environment in the different compounds and showing which doping sites are the most effective at improving the thermoelectric properties, we hope this study will be helpful for future experimental work. Our theoretical study can be expanded to substitutions at the anionic sites with other elements than As atoms. In addition, using a similar approach, the study can be extended to substitutions at the cationic sites as well.
